# Distinct pressure half-time values by transthoracic echocardiography for grading of paravalvular regurgitation after transcatheter aortic valve replacement

**DOI:** 10.1038/s41598-020-59211-z

**Published:** 2020-02-13

**Authors:** Joerg Schröder, Mohammad Almalla, Mohammed Saad, Matthias Mezger, Andras Keszei, Michael Frick, Shahram Lotfi, Rainer Hoffmann, Michael Becker, Ertunc Altiok

**Affiliations:** 1 Department of Cardiology, Angiology and Intensive Care, University Hospital RWTH Aachen, Aachen, Germany; 2 Department of Medicine - Cardiology, Angiology, Intensive Care Medicine, University Hospital Lübeck, Lübeck, Germany; 3 Department of Medical Informatics, University Hospital RWTH Aachen, Aachen, Germany; 4 Department of Cardiovascular Surgery, University Hospital RWTH Aachen, Aachen, Germany; 5 Department of Cardiology, St. Bonifatius Hospital, Lingen, Germany; 6 Department of Cardiology, Nephrology and Internal Intensive Care Medicine, Rhein-Maas Klinikum, Wuerselen, Germany

**Keywords:** Interventional cardiology, Echocardiography

## Abstract

Postprocedural aortic regurgitation (AR) has negative impact on patient outcome after transcatheter aortic valve replacement (TAVR). Standard assessment of AR severity by echocardiography is hampered after TAVR. Measurement of pressure half-time (PHT) by echocardiography is not limited in these patients but it may be affected by concomitant left ventricular hypertrophy (LVH). This study sought to evaluate distinct cut-off values of PHT differentiating between patients without and with more than mild LVH for grading of AR after TAVR with cardiac magnetic resonance (CMR) as the reference method for comparison. 71 patients (age 81 ± 6 years) with severe aortic stenosis undergoing TAVR were included into the study. Transthoracic echocardiography (TTE) and CMR were performed after TAVR. Left ventricular mass index was calculated by TTE. PHT was measured by continuous-wave Doppler echocardiography of aortic regurgitation jet. In 18 patients (25%) PHT could not be obtained due to no or very faint Doppler signal. Aortic regurgitant volume and regurgitant fraction were calculated by CMR by flow analysis of the ascending aorta. In 14 of 53 patients (26%) AR after TAVR was moderate or severe as categorized by CMR analysis. More than mild LVH was present in 27 of 53 patients (51%). PHT correlated inversely less to regurgitant fraction by CMR analysis in patients with LVH (r = −0.293; p = 0.138) than in patients without LVH (r = −0.455; p = 0.020). In patients without relevant LVH accuracy of PHT to predict moderate or severe paravalvular regurgitation AUC was 0.813 using a cut-off value of 347 ms and AUC was 0.729 in patients with more than mild LVH using a cut-off value of 420 ms. Analysis of PHT by TTE with distinct cut-off values for patients without and with more than mild LVH allows detection of moderate or severe AR after TAVR as defined by CMR. In none of the patients in which PHT could not be measured AR was categorized as more than trace by CMR analysis.

## Introduction

Transcatheter aortic valve replacement (TAVR) has become an alternative to conventional operation in patients with severe symptomatic aortic valve stenosis^1–4^. Postprocedural aortic regurgitation (AR) has negative impact on patient outcome after TAVR^2,3,5–11^. Transcatheter heart valve prostheses are implanted in a sutureless technique and regurgitation is in most cases caused by insufficient sealing between prosthesis and aortic ring^12^. Grading of AR after TAVR has substantial limitations using transthoracic echocardiography (TTE) due to paravalvular localization of regurgitation. Therefore, echocardiographic criteria for quantification of AR severity after TAVR have been defined by the valve academic research consortium (VARC)^13^. However, most of the measurements used in the VARC II-guidelines are approved for quantification of native valve regurgitation, and no recommendations are provided in case of discrepancy in AR severity grouping by the different parameters. Pressure half-time (PHT) is a parameter which can be simply obtained by TTE, but it is influenced by elevated filling pressures mostly in case of left ventricular hypertrophy (LVH) which is common in patients with aortic stenosis^14^. Cardiac magnetic resonance (CMR) has become established for precise quantification of valvular regurgitation^15^.

This study sought to evaluate the accuracy of PHT with distinct cut-off values separating patients without and with more than mild LVH for grading of paravalvular AR after TAVR in comparison with cardiac magnetic resonance (CMR) as the reference method.

## Methods

**Figure 1:**
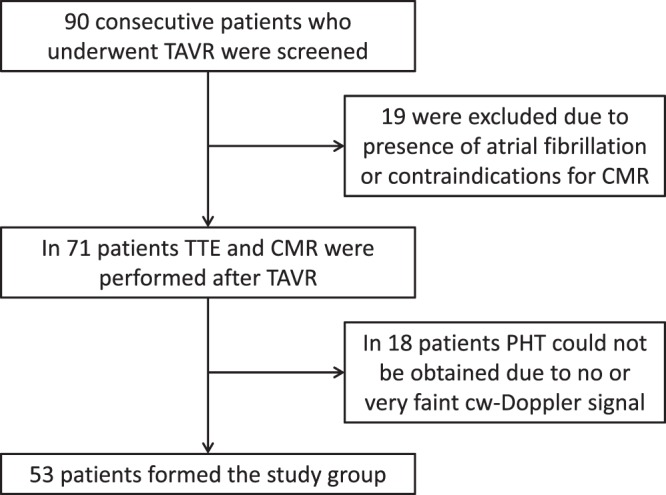
Patient selection chart.

From 2010 to 2014, 90 consecutive patients with severe aortic stenosis undergoing TAVR either by the CoreValve system (Medtronic, Minneapolis, Minnesota) or the Edwards SAPIEN XT valve (Edwards Lifesciences, Irvine, California) were screened and 19 patients were excluded due to presence of atrial fibrillation or contraindications for CMR (all due to device therapy). In the remaining 71 patients (age 81 ± 6 years) TTE and CMR were performed after TAVR (Fig. 1). This study was approved by the Ethics committee of the Faculty of medicine, University RWTH Aachen. All research was performed in accordance with the relevant guidelines and regulations. Informed consent was obtained from all participants or their legal guardians (NCT 01966146).

### Transthoracic echocardiography

**Figure 2:**
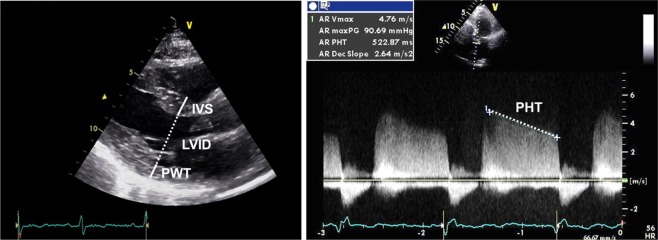
Echocardiographic parasternal long-axis view with linear measurements of the left ventricle performed at end-diastole. Left ventricular mass was calculated by the formula: LV mass = 0.8*1.04*[(IVS + LVID + PWT)³−LVID³] + 0.6 g where IVS is interventricular septum, LVID is LV internal diameter, and PWT is inferolateral wall thickness (left panel). Pressure half-time (PHT) was measured by analysis of the continuous-wave Doppler velocity curve of aortic regurgitation jet (right panel).

Echocardiographic studies were performed with a commercially available echocardiographic system (Vivid E9, General Electric, Vingmed, Horton, Norway) and 2D transthoracic probe (M5S). Echocardiographic parasternal long-axis view was acquired for linear measurements of the left ventricle at end-diastole. Left ventricular (LV) mass was calculated by the ASE/EAE formula using linear transthoracic echocardiographic dimensions: *LV mass* = *0*.*8* * *1*.*04* * *[(IVS* + *LVID* + *PWT)³* − *LVID³]* + *0*.*6* *g* where IVS is interventricular septum, LVID is LV internal diameter, and PWT is inferolateral wall thickness (Fig. 2, left). LVH was assumed when LV mass index was more than mildly abnormal (>108 g/m² in women and >131 g/m² in men), according to recommendations by the American Society of Echocardiography and the European Association of Cardiovascular Imaging^16^. PHT was measured by analysis of continuous-wave Doppler curve of aortic regurgitation jet from an apical 3-chamber or apical 5-chamber view in diastole (Fig. 2, right). In 18 of 71 patients (25%) with at most trace regurgitation, PHT could not be obtained due to no or very faint signal. Regurgitation volume was calculated as the difference between stroke volume in the left ventricular outflow tract (LVOT) and pulmonary flow in systole assessed by pulsed-wave Doppler echocardiography. Regurgitant fraction was calculated by dividing regurgitant volume by stroke volume in the LVOT as previously described^17^.

### Cardiac magnetic resonance

**Figure 3:**
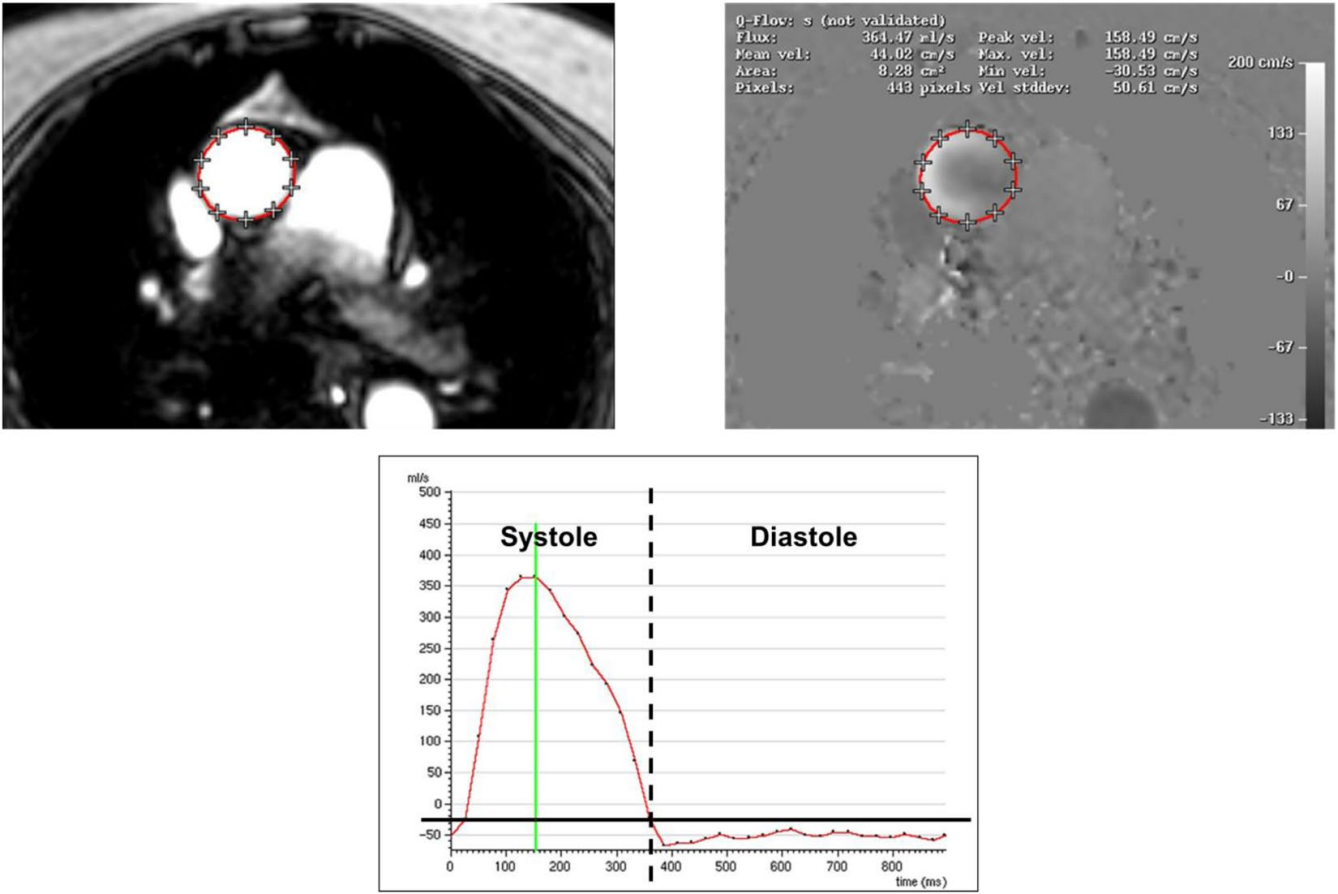
Quantitative flow images by CMR acquired in the ascending aorta (upper panels) with measurement of the aortic flow in diastole for quantification of regurgitation volume and fraction after TAVR (lower panel).

CMR was applied on a 1.5 Tesla MR-scanner (Achieva, Philips Healthcare, Best, The Netherlands) equipped with a 5-element cardiac synergy coil for signal reception and a vector-ECG for cardiac synchronization. Based on survey- and standard cine-imaging, a through plane velocity encoded phase-contrast sequence (Q-flow; 35 phases per cardiac cycle; spatial resolution 1.4 × 1.4 × 10 mm; TR/TE/flip angle: 3.9 ms/2.4 ms/15o; breath hold duration 12–18 seconds) was planned orthogonal to the ascending aorta just above the cage of the TAVR prosthesis. Maximum velocity encoding was adapted individually to avoid aliasing. Quantitative analysis was performed offline on a dedicated MR-workstation (Extended Workspace, Philips Healthcare, Best, The Netherlands). Regurgitant volume was derived by measurement of diastolic aortic backward flow. Regurgitant fraction was calculated by division of aortic backward flow by aortic forward flow as previously described (Fig. 3)^17^. According to CMR recommendations, paravalvular regurgitation severity was graded more as trace when regurgitant fraction was <8%, as mild when regurgitant fraction was 8% to 19%, as moderate when regurgitant fraction was 20% to 29% and severe when regurgitant fraction was >29%^18^.

### Statistical analysis

Statistical analysis was performed with dedicated analysis program (MedCalc Software, Version 19.1, Ostend, Belgium). Continuous data are presented as mean ± standard deviation. The Pearson correlation coefficient (r) was calculated to evaluate agreement between PHT by TTE and regurgitant fraction by CMR, and relationship was visualized by scatter diagram with regression lines differentiated for the presence or absence of LVH. 95% confidence interval (CI) was reported where adequate. Analysis of variance (ANOVA) analysis was performed for PHT by TTE as graded by CMR regurgitant fraction. Student-Newman-Keuls test was used for post-hoc pairwise comparisons of PHT by TTE as graded by CMR regurgitant fraction. Cut-off values of PHT were defined as the optimal criterions corresponding with the Youden index for differentiating trace or mild from moderate or severe AR as graded by CMR regurgitant fraction. Intraclass correlation coefficient (ICC) was calculated for assessment of interobserver and intraoserver agreement. A p < 0.05 was considered significant.

## Results

Patient characteristics are given in Table 1. Echocardiographic and CMR measurements of AR after TAVR are displayed in Table 2. In 53 of 71 patients (75%) measurement of PHT was possible while in 18 patients (25%) PHT could not be obtained due to no or very faint signal of continues-wave Doppler. In patients PHT could not be measured, regurgitant volume was 5.0 ± 2.2 ml (range from 3.1 to 8.7 ml) and regurgitant fraction was 7.8 ± 3.6% (range from 1% to 15%) by CMR analysis after TAVR. Further results included the remaining 53 patients in whom PHT could be measured. Of these 53 patients, 27 (51%) had more than mild LVH (LV mass index = 159 ± 41 g/m²) and 26 patients (49%) had at most mild LVH (106 ± 16 g/m²). Based on CMR regurgitant fraction, AR severity after TAVR was graded as trace in 13 of 53 patients (25%), mild in 26 patients (49%), moderate in 8 patients (15%) and as severe in 6 patients (11%). In patients without and with LVH lower PHT values (indicating higher degree of regurgitation) were seen in AR graded as moderate to severe compared to AR graded as trace or mild by CMR regurgitant fraction analysis (p = 0.087 and p = 0.147; respectively) (Table 3).

**Table 1:** Patient characteristics.

Variable	n = 71
Age, years	81 ± 6
Men, n	32 (45%)
Edwards SAPIEN XT, n	39 (55%)
CoreValve, n	32 (45%)
Logistic Euroscore, %	21 ± 14
Ejection fraction, %	52 ± 12
Left ventricular mass, g	229 ± 76
Left ventricular mass index, g/m²	131 ± 40
**NYHA functional classification**, **n**	
I	1 (1%)
II	9 (13%)
III	50 (70%)
IV	11 (16%)
Diabetes mellitus, n	23 (32%)
Hypertension, n	58 (81%)
Hypercholesterolemia, n	48 (67%)
Renal insufficiency, n	26 (36%)
Smoker, n	15 (21%)

NYHA: New York Heart Association.

**Table 2:** Echocardiographic and cardiac magnetic resonance measurements of aortic regurgitation after transcatheter aortic valve replacement.

Variable	PHT not obtained (n = 18)*	PHT study group (n = 53)	p-value
**Transthoracic echocardiography**			
Paravalvular aortic jet length (cm)	n.a.	1.6 ± 1.3	n.a.
Pressure half-time (ms)	n.a.	529 ± 183	n.a.
Aortic regurgitant volume (ml)	3.8 ± 3.9	10.3 ± 10.1	p = 0.010
Aortic regurgitant fraction (%)	6.8 ± 6.1	16.2 ± 11.7	p = 0.002
**Cardiac magnetic resonance**			
Aortic regurgitant volume (ml)	5.0 ± 2.2	10.7 ± 10.6	p = 0.025
Aortic regurgitant fraction (%)	7.8 ± 3.6	15.1 ± 11.2	p = 0.008

*PHT could not be obtained in 18 of 71 patients due to no or very faint Doppler signal.

Values are presented in mean ± standard deviation. Significance level by one way analysis of variance (ANOVA).

PHT: pressure half-time.

**Table 3:** PHT of aortic regurgitation after TAVR assessed by transthoracic echocardiography in all patients of the study group in which PHT could be measured* (n = 53 of 71 patients), and differentiated between patients without (n = 26) and with (n = 27) more than mild LVH related to regurgitation grade as determined by CMR analysis of regurgitant fraction.

Variable	Trace	Mild	Moderate to severe	p-value
**Grading of aortic regurgitation severity by CMR**				
PHT in all patients of the study group* (ms)	582 ± 213 (n = 13)	570 ± 154 (n = 26)	404 ± 153^#^ (n = 14)	p = 0.009
PHT in patients without LVH (ms)	587 ± 239 (n = 10)	612 ± 162 (n = 12)	351 ± 187 (n = 4)	p = 0.087
PHT in patients with LVH (ms)	565 ± 117 (n = 3)	532 ± 144 (n = 14)	425 ± 143 (n = 10)	p = 0.147

*PHT could not be obtained in 18 of 71 patients due to no or very faint Doppler signal.

^#^p < 0.05 by Student-Newman-Keuls test for all pairwise comparisons: PHT in moderate to severe vs. trace or mild aortic regurgitation by CMR.

Values are presented in mean ± standard deviation.

CMR: cardiac magnetic resonance, LVH: left ventricular hypertrophy, PHT: pressure half-time, TAVR: transcatheter aortic valve replacement.

PHT inversely correlated low to regurgitant volume (r = −0.416, 95% CI −0,617 to −0,165; p = 0.002) and regurgitant fraction by CMR analysis in all patients (r = −0.401, 95% CI −0,606 to −0,147; p = 0.003). Regarding only patients without LVH, PHT correlated more to regurgitant volume by CMR analysis (r = −0.438, 95% CI −0,705 to −0,061; p = 0.025) compared to analysis in patients with LVH (r = −0.357, 95% CI −0,649 to 0,026; p = 0.067). Similarly, regarding patients without LVH, PHT correlated more to regurgitant fraction by CMR analysis (r = −0.455, 95% CI −0,716 to −0,082; p = 0.020) compared to analysis in patients with LVH (r = −0.293, 95% CI −0,606 to 0,098; p = 0.138).

**Figure 4:**
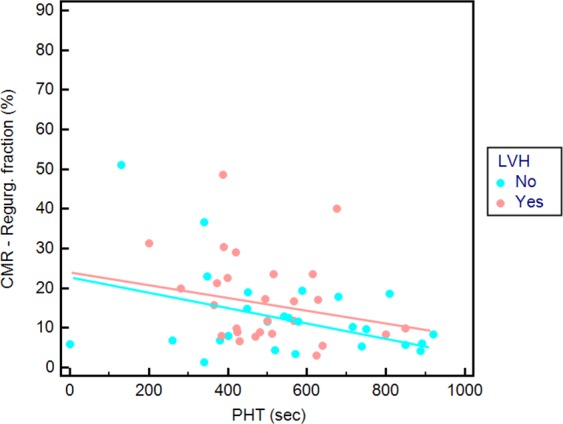
Scatter diagram of pressure half-time (PHT) by transthoracic echocardiography and aortic regurgitant fraction by cardiac magnetic resonance (CMR) of paravalvular regurgitation after TAVR (n = 53 patients in which PHT could be measured) differentiated between patients without (blue dots; n = 26) and with (red dots; n = 27) more than mild left ventricular hypertrophy (LVH).

Relationship of PHT and CMR regurgitant fraction was visualized by scatter diagram with distinguished regression lines in patients with presence (intercept = 28.3, slope = −0.022; p = 0.138) and absence of LVH (intercept = 26.1, slope = −0.023; p = 0.002) (Fig. 4).

In patients without LVH, accuracy of PHT to predict moderate to severe AR as defined by CMR regurgitant fraction using a cut-off value of 347 ms was numerically higher (AUC = 0.813, 95% CI 0,533 to 1,000; sensitivity 75.0%, specificity 90.9%) than in patients with LVH using a cut-off value of 420 ms (AUC = 0.729, 95% CI 0,504 to 0,954; sensitivity 70.0%, specificity 88.2%). Looking at all patients, 77% could be correctly classified having more than mild AR or not by PHT assessment using a cut-off value of 420 ms. Additional analysis differentiating patients with and without LVH using distinct PHT cut-off values leaded to an increase to 81% of correctly identified cases as graded by CMR regurgitant fraction. However, improvement of accuracy using different cut-off values in patients with and without LVH did not reach statistical significance (p = 0.6499).

There was high intraobserver and interobserver agreement of re-measurements of PHT in all patients (ICC = 0.938, 95% CI 0.895 to 0.9639 and ICC = 0.871, 95% CI 0.786 to 0.924; respectively).

## Discussion

The major findings of this study are that (1) PHT values assessed by TTE are different in patients with trace or mild and moderate to severe AR after TAVR as graded by CMR regurgitant fraction, (2) accuracy of PHT for differentiation between trace or mild and moderate to severe AR was better in patients without LVH compared to patients with LVH and (3) definition of different cut-off values of PHT for patients with and without LVH tended to improve detection of patients with moderate to severe AR after TAVR as defined by CMR regurgitation fraction as the reference method.

Quantification of AR by TTE has particularly been validated in native valves with commonly central regurgitant jets and grading after TAVR has considerable limitations due to paravalvular localization of regurgitation^12^. Therefore VARC II-criteria for grading of AR severity by TTE have been proposed including semi-quantitative parameters with evaluation of diastolic flow reversal in the descending aorta and quantification of circumferential extent of prosthetic valve paravalvular regurgitation as well as quantitative parameters with quantification of the prosthetic regurgitant volume, regurgitant fraction and calculation of effective regurgitant orifice area^13^. However, in case of deviances between the five proposed parameters in the categorization of AR severity, there is no distinct path on bringing the divergent measurements to a combined result. In particular, no parameter has been defined, which should be the foremost parameter for categorization of AR severity in case of divergence. Moreover, variations and overlaps of different grading scales result in confusion. Therefore, an even more extended graduation with a 5-class scheme was proposed making evaluation of regurgitation severity after TAVR more complex^19^.

Even in native valvular disease, grading of AR severity has been demonstrated to be limited by a significant interobserver variability in comparison to CMR analysis if common TTE parameters for AR assessment being part of the VARC II-criteria are applied^20^. Recently, interobserver agreements of the VARC II quantitative parameters regurgitant volume, regurgitant fraction and calculation of effective regurgitant orifice area by TTE after TAVR have been shown to be low (ICC = 0.59, 0.61 and 0.47, respectively) with high variation (coefficient of variation = 0.67, 0.82, 0.54, respectively) compared to assessment of PHT (ICC = 0.73, coefficient of variation = 0.10)^21^. This corresponds to the high interobserver and intraobserver agreement of PHT in our study in which CMR as a more precise technique and not angiography was the reference method. The same authors investigated VARC-II criteria and additional TTE parameters in another study for grading of AR severity after TAVR. They found out that only in 58% of patients more than mild AR could be identified by VARC II-criteria. Even such a simply assessable parameter like PHT had better accuracy (AUC = 0.66, sensitivity = 75%, specificity = 62%) for detecting more than mild AR than complex analysis with VARC II-criteria (AUC = 0.63, sensitivity = 75%, specificity = 52%)^22^. But in that study, PHT cut-off value of 403 ms was different from the recommended cut-off value of 500 ms in current echocardiography guidelines for definition of more than mild AR in native valve disease^23^. The authors assumed abnormal aortic compliance and particularly LVH leading to flow characteristics different from chronic AR as a reason for the differing PHT cut-off value. The limitation of that study was that they used angiographic assessment of AR as reference method which provides a subjective qualitative grading and may inconsistently correlate with quantitative assessment of AR^24^. In a study of our institution we found similar results that VARC II-criteria by TTE had only moderate accuracy in AR severity grading when compared with CMR imaging as a quantitative reference method (Kappa = 0.357)^17^.

PHT is known to be a parameter for AR quantification by TTE which is mainly influenced by elevated filling pressures in case of LVH^14^. In patients with aortic stenosis, LVH is common^25^. We assumed that differences in extent of LVH, which is similar easy to assess by TTE like PHT, may result in different filling pressures. Therefore, we differentiated patients with at most mild LVH (49%) from patients with moderate to severe LVH (51%) according to criteria recommended by the American Society of Echocardiography and the European Association of Cardiovascular Imaging^16^. In 25% of patients, PHT could not be obtained due to no or very faint signal of regurgitation jet. In these patients regurgitant fraction was 7.8 ± 3.6% indicating only trace AR by CMR analysis. Conveniently, in none of these patients more than mild AR as defined by CMR analysis with regurgitation fraction of at most 15% (CMR definition of moderate AR when regurgitant fraction was >19%) was observed^18^. Both PHT cut-off values of 347 ms and 420 ms for patients without and with more than mild LVH were lower than recommendation of PHT cut-off value by the American Society of Echocardiography and the European Association of Cardiovascular Imaging recommendations^23^. Our study using CMR analysis as reference method supports the results of a previous study demonstrating that in patients after TAVR, lower PHT cut-off value has to be applied for detecting more than mild AR using angiographic assessment as the reference method^22^. According to that study where 68% of patients after TAVR could be correctly categorized having more than mild AR as defined by angiographic assessment in our study 77% of patients could be correctly categorized having more than mild AR or not as defined by CMR analysis using one PHT cut-off value for all patients. Using distinct PHT values for patients with and without LVH has leaded to an increase of correct categorization in 81% of cases. Disappointingly, improvement of accuracy by differentiating between patients with and without LVH was not statistically significant. This may be explained by the small number of patients in our study. Furthermore, elevated filling pressure reduces accuracy of PHT analysis in patients with AR^14^. In patients with LVH higher filling pressures have to be expected compared to patients without relevant LVH. As expected, accuracy of PHT for detection of more than mild AR as graded by CMR analysis tended to be better in patients without LVH. A study with a larger number of patients or additional parameters directly assessing left ventricular filling pressures may be needed to better support these findings.

Different rates of AR have been reported after TAVR with the reported frequency of moderate to severe regurgitation up to 24% in early generation prostheses. The number of patients with moderate or severe AR after TAVR in our study (20%) was in line with previous reports using either by the CoreValve system or the Edwards SAPIEN XT valve^2–7^. Newer generation of TAVR prostheses have been developed with incrementally lower grades of moderate or severe AR^26^. Less occurrence of more than mild regurgitation after TAVR may reduce the relevance of AR quantification by TTE. Moreover, therapeutic consequences of detection of significant AR after TAVR are limited^19^. However, accurate grading of AR after TAVR has at least prognostic implications because even moderate AR is associated with negative impact on patient outcome^5–11^.

## Conclusions

Analysis of PHT as an easy approach by TTE with distinct cut-off values for patients without and with more than mild LVH allows identification of moderate or severe AR after TAVR as defined by CMR as the reference method. In a quarter of the patients PHT could not be measured by TTE due to no or very faint Doppler signal. In none of these patients AR was categorized as more than trace by CMR analysis.
